# Protein kinase Msk1 physically and functionally interacts with the KMT2A/MLL1 methyltransferase complex and contributes to the regulation of multiple target genes

**DOI:** 10.1186/s13072-016-0103-3

**Published:** 2016-11-11

**Authors:** Maaike Wiersma, Marianne Bussiere, John A. Halsall, Nil Turan, Robert Slany, Bryan M. Turner, Karl P. Nightingale

**Affiliations:** 1 Chromatin and Gene Expression Group, Institute of Cancer and Genomic Sciences, College of Medical and Dental Sciences, University of Birmingham, Birmingham, B15 2TT UK; 2 Institute of Clinical Sciences, College of Medical and Dental Sciences, University of Birmingham, Birmingham, B15 2TT UK; 3 Molecular Imaging and Photonics, KU Leuven, Celestijnenlaan 22f, Box 2404, 3001 Louvain, Belgium; 4 Department of Biology, Friedrich-Alexander-University of Erlangen-Nürnberg, 91058 Erlangen, Germany

**Keywords:** MLL1 complex, Chromatin, Histone modification, Signal transduction, Gene regulation

## Abstract

**Background:**

The KMT2A/MLL1 lysine methyltransferase complex is an epigenetic regulator of selected developmental genes, in part through the SET domain-catalysed methylation of H3K4. It is essential for normal embryonic development and haematopoiesis and frequently mutated in cancer. The catalytic properties and targeting of KMT2A/MLL1 depend on the proteins with which it complexes and the post-translational protein modifications which some of these proteins put in place, though detailed mechanisms remain unclear.

**Results:**

KMT2A/MLL1 (both native and FLAG-tagged) and Msk1 (RPS6KA5) co-immunoprecipitated in various cell types. KMT2A/MLL1 and Msk1 knockdown demonstrated that the great majority of genes whose activity changed on KTM2A/MLL1 knockdown, responded comparably to Msk1 knockdown, as did levels of H3K4 methylation and H3S10 phosphorylation at KTM2A target genes *HoxA4*, *HoxA5*. Knockdown experiments also showed that KMT2A/MLL1 is required for the genomic targeting of Msk1, but not vice versa.

**Conclusion:**

The KMT2A/MLL1 complex is associated with, and functionally dependent upon, the kinase Msk1, part of the MAP kinase signalling pathway. We propose that Msk1-catalysed phosphorylation at H3 serines 10 and 28, supports H3K4 methylation by the KMT2A/MLL1 complex both by making H3 a more attractive substrate for its SET domain, and improving target gene accessibility by prevention of HP1- and Polycomb-mediated chromatin condensation.

**Electronic supplementary material:**

The online version of this article (doi:10.1186/s13072-016-0103-3) contains supplementary material, which is available to authorized users.

## Background

The human KMT2 proteins (formerly known as mixed-lineage leukaemia, MLL proteins) are highly conserved, SET domain-containing lysine methyltransferases, homologous to the *Drosophila* trithorax group proteins TRX (trithorax), TRR (TRX-related) and dSET1 [1, 2]. Like their *Drosophila* homologues, they maintain and enhance the activity of genes with key roles in differentiation and development [2], in part by regulating levels of histone 3 lysine 4 (H3K4) methylation, thereby, directly or indirectly, modifying chromatin structure [3, 4]. In humans, the KMT2 family comprises three pairs of paralogues, namely KMT2A and 2B (dTRX homologues, originally MLL1 and 2), KMT2C and 2D (dTRR homologues, originally MLL3 and 4) and KMT2F and G (dSET1 homologues, originally hSET1A and hSET1B). KMT2 family members are frequently mutated in leukaemias [5] and other cancers [6]. In vivo, the KMT2 enzymes are found in multi-subunit complexes of which three proteins, WDR5, RbBP5 and ASH2L, provide a common stoichiometric core (reviewed in [1, 7]). These proteins enhance the weak catalytic activity of recombinant KMT2 by 50–500-fold [8, 9]. DPY30 also frequently associates with the core complex, providing a further twofold increase in methyltransferase activity [9]. In addition to these common components, complexes built on each one of the three KMT2 subgroups can interact with a unique set of binding proteins. For example, only KMT2A/B complexes contain multiple endocrine neoplasia type 1 (MENIN) [10] and lens epithelium-derived growth factor (LEDGF) [11]. These proteins seem to mediate interaction with transcription factors such as oestrogen receptor [12] and therefore influence gene targeting. In addition, KMT2 complexes have been shown to interact dynamically with a variety of transcription factors, including E2Fs [13] and p53 [14] amongst several others (see [1, 7] for further details). It is likely that these variable protein associations make a major contribution to the catalytic properties and gene regulation profiles of the three KMT2 subgroups.

All KMT2 subgroups are specific for H3K4, but catalyse different methylation states. Recombinant KMT2A/B complexes give mono- and di-methylation (H3K4me1, H3K4me2), with only weak trimethylation [1, 15, 16]. KMT2C/D complexes give predominantly mono-methylation, whilst KMT2F/G complexes are capable of mono-, di- and trimethylation [1]. The distinction is important, given that the different methylation states of H3K4 play different roles in transcriptional control. H3K4me1 is a consistent marker of regulatory enhancers [17, 18], whereas H3K4me3 is, amongst other things, a marker of active promoter regions [19, 20]. Various factors could contribute to these detailed, but functionally important, catalytic differences. For example, H2B ubiquitination enhances the activity of KMT2A/MLL1 and KMT2F, but not KMT2C [16]. In addition, post-translational modification of individual subunits can influence catalytic properties; for example, SUMOylation of RbBP5 inhibits KMT2A/MLL1 by disrupting its association with ASH2L [21]. These events may account for the occasional discrepancies between the catalytic properties of purified complexes and those reconstituted in vitro from recombinant proteins (see [1] for original references). Thus, much remains to be learned about the ways in which interacting factors and post-translational protein modifications determine the characteristic properties of particular KMT2 complexes. As the catalytic and structural requirements for the KMT2 complex are likely to be context dependent, varying from one cell type or developmental stage to another, any individual cell may contain a dynamic and variable population of complexes with different protein components.

The results presented here focus on the most widely studied mammalian KMT2, namely the TRX homologue KMT2A/MLL1. Human KMT2A is a large protein, 3963 amino acids which, uniquely within the KMT2 family, contains two sites that can be cleaved by the threonine aspartase Taspase 1 [22, 23]. (Its paralogue, KMT2B, contains only one such site.) Taspase 1 cleavage of newly translated KMT2A/MLL1 generates 320 kDa N-terminal and 180 kDa C-terminal fragments [24], which then form a non-covalent, heterodimeric complex. How this cleavage step contributes to KMT2A/MLL1 function remains uncertain [25], but it is likely that it allows the more effective configuration of the various functional domains spread across the protein. These include the lysine methyltransferase (SET) domain [3, 19]), and various protein-binding (PHD, bromo-domains [26]) and DNA-binding (CXXC, AT hook) domains [27, 28]). After post-translational processing, KTM2A/MLL1 is incorporated into a functional, multi-protein complex containing the four core components common to all KMT2 complexes (outlined above), plus a variable number of additional components [29]. Some of these confer specific catalytic properties, including the lysine acetyltransferase CBP/KAT3A, which is necessary for the complex to activate transcription [30, 31], and MOF/KAT8, which specifically acetylates lysine 16 on histone H4, a modification known to decondense chromatin [32].

The association of histone acetyltransferases with KMT2A complexes and the effect of H2B ubiquitination on its methyltransferase activity exemplify the complex interactions (‘cross-talk”) of histone post-translational modifications (PTMs) as determinants of chromatin function. We have shown previously that global histone hyperacetylation induced by histone deacetylase inhibitors is accompanied, in at least some cell types, by increased methylation of H3K4 [33, 34]. In an attempt to explain this, we asked how PTMs might affect the ability of the H3 Tail to act as a methyltransferase substrate. In vitro assays using a recombinant SET domain methyltransferase and synthetic peptide substrates showed that H3 mono-, di- and trimethylation were maximally enhanced by acetylation of both H3K9 and H3K14, together with phosphorylation of H3S10 [33]. Here, we address the in vivo significance of H3S10 phosphorylation for function of the KMT2A methyltransferase complex and examine the mechanism by which this modification is put in place. We show that mitogen- and stress-activated kinase, Msk1 (also known as RPS6KA5), is physically associated with the KMT2A/MLL1 complex in vivo and is necessary for the regulation of key KMT2A-regulated genes.

## Results

### KMT2A and Msk1 are physically associated in various cell lines

**Fig. 1:**
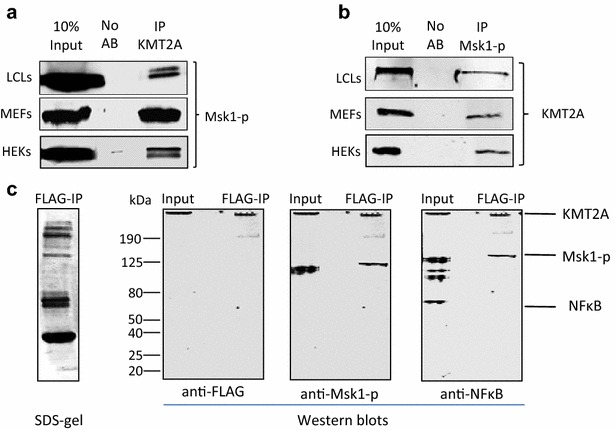
Co-immunoprecipitation of KMT2A and Msk1. **a** Western blots analysing KMT2A-immunoprecipitated material from human lymphoblastoid cells (LCLs), mouse embryonic fibroblasts (MEFs) and human embryonic kidney cells (HEKs). The precipitated material tested positively for Msk1. A representative western (1 out of 3) is shown. **b** Western blots analysing Msk1-immunoprecipitated material from the indicated cell lines. The precipitated material tested positively for KMT2A. A representative western (1 out of 3) is shown. **c** FLAG-KMT2A was overexpressed in HEK293 cells, immunoprecipitated using FLAG antibodies and characterised by SDS gel (silver nitrate stain, *left panel*). The gel was Western-blotted and the filter-stained sequentially with antibodies to FLAG, phosphorylated Msk1 and NFκB as indicated

The in vitro methylation of H3K4 by the KMT2A/MLL1 SET domain is strongly enhanced by phosphorylation of the histone peptide substrate at serine 10 [33], raising the possibility that phosphorylation of this residue may influence the gene regulatory activity of the KMT2A/MLL1 complex in vivo. Phosphorylation of H3S10 in chromatin can occur through the action of the closely related kinases Msk1 and Msk2 [35, 36], so we explored the possibility that Msk1 might operate as part of the KMT2A/MLL1 complex. Msk1 is constantly expressed in the cells but is active only after (auto-) phosphorylation and allosteric changes induced by ERK1 or 2 [37]. The antibody used for these experiments is specific for Msk1 phosphorylated at S360, one of the last residues to become phosphorylated, and detects active Msk1 [38]. In three different cell types—human lymphoblastoid cells (LCLs), mouse embryo fibroblasts (MEFs) and human embryonic kidney cells (HEKs)—material precipitated with antibodies to KMT2A contained readily detectable amounts of Msk1 (Fig. 1a). The protein often migrated as a double band, possibly due to allosteric changes and/or differences in phosphorylation status (Fig. 1a). Conversely, antibodies to Msk1 immunoprecipitated KMT2A/MLL1 (Fig. 1b, full-length blots from 1A and 1B are shown in Additional file 1). To validate this interaction, FLAG-tagged KMT2A/MLL1 was overexpressed in HEK293 cells and the associated complex purified (Fig. 1c). Western blot analysis of the immunoprecipitated complex confirmed that it contained the FLAG-tagged KMT2A/MLL1 and also Msk1, but not NFκB, a known interaction partner of Msk1 [39] readily detected in the lysate (Fig. 1c). It seems that the KMT2A/MLL1–Msk1 interaction is specific, stable and present in a range of mouse and human cell types.

### KMT2A and Msk1 knockdowns trigger similar expression changes in KMT2A target genes

**Fig. 2:**
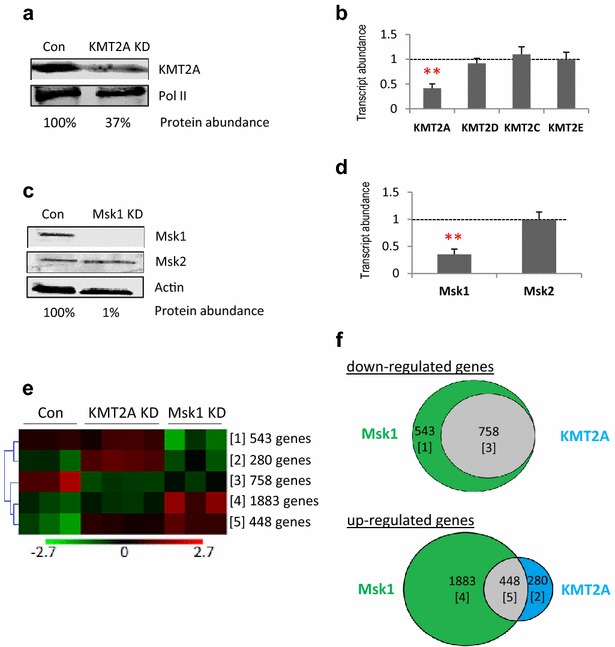
Effects of KMT2A/MLL1 and Msk1 knockdown on *HoxA* gene expression. **a** Western analysis of KMT2A/MLL1 abundance in mouse embryo fibroblasts (MEFs), either mock-transfected (Con) or KMT2A/MLL1 knockdown (KD). RNA polymerase II (*Pol II*) was used as a loading control and to calculate the extent of KMT2A/MLL1 knockdown (37% of control). Full-length blots are given in Additional file 1. **b** RT-qPCR expression analysis of a selection of KMT2 genes in knockdown cells using actin as a control and with mock-transfected cells normalised to 1.0 (*n* = 3, *T* test, *p < 0.05; **p < 0.01; ***p < 0.001). The KMT2E protein has no methyltransferase activity (see text). **c** Western analysis of Msk1 and Msk2 in mock-transfected (Con) and Msk1 knockdown (KD) MEFs. Actin was used as a loading control and to calculate the extent of Msk1 knockdown (1% of control). A representative western (1 out of 3) is presented. Full-length blots are given in Additional file 1. **d** RT-qPCR expression analysis of Msk1 and 2 in Msk1 knockdown cells. Transcripts were analysed using actin as a control, with the level of transcripts in mock-transfected cells normalised to 1.0 (*n* = 3, *T* test, *p < 0.05; **p < 0.01; ***p < 0.001). **e** Relative gene expression levels in control (*n* = 3), KMT2A/MLL1 knockdown (*n* = 4) and Msk1 knockdown (*n* = 3) cells are indicated by colour intensity from 2.7-fold down-regulated (*green*) to 2.7-fold up-regulated (*red*). 3912 genes showed significant changes in expression following KMT2A/MLL1 and/or MSK1 knockdown and these were clustered into five groups by SOTA analysis; the number of genes in each cluster is indicated. **f** Venn diagrams showing the numbers and relationship of genes down-regulated (*upper panel*) or up-regulated (*lower panel*), by knockdown of KMT2A/MLL1 and/or Msk1. The corresponding SOTA cluster is indicated in *brackets*

We analysed the possible functional impact of the KMT2A/MLL1–Msk1 interaction by using a knockdown approach in MEFs. These cells express *HoxA* genes, known KMT2A/MLL1 targets [40]. Transient siRNA-mediated knockdown conditions were established for KMT2A/MLL1, resulting in a >40% reduction in the protein (Fig. 2a) that was well tolerated by the cells. The knockdown was specific, with no impact on the transcripts of other mouse KMT genes (Fig. 2b). For Msk1, we established well-tolerated knockdown conditions that reduced Msk1 protein abundance by >90% (Fig. 2c), whilst leaving protein and transcript levels of its close paralogue Msk2 unaffected (Fig. 2c, d).

We used expression analysis on high-density microarrays to identify genes whose expression was sensitive to knockdown of KMT2A/MLL1 or Msk1. Using a fold change cut-off of 2 and false discovery rate <10%, 3912 different genes changed expression in response to either KMT2A/MLL1 or Msk1 knockdown. Undirected cluster analysis (SOTA) separated the responding genes into five groups, as shown in Fig. 2e. The up- and down-regulation of selected genes was validated by RT-qPCR (Additional file 2).

Strikingly, all 758 genes that were significantly down-regulated in response to the loss of KMT2A/MLL1 [cluster 3 genes] were also down-regulated in response to the loss of Msk1 (Fig. 2f, upper panel). Thus, *all* genes whose ongoing transcriptional activity is dependent on KMT2A/MLL1 (i.e. that are *down*-*regulated* by KMT2A KD) are also dependent on Msk1. The overlap of responding genes was much greater than expected by chance (Chi-squared test with Yates correction, two-tailed *p* value <0.0001), indicating a functional relationship between the two enzymes. This 100% overlap is not attributable to down-regulation of Msk1 in KMT2A/MLL1 KD cells or vice versa. Expression of KMT2A/MLL1 in Msk1 knockdown cells was not significantly altered, whereas the expression of Msk1 showed a modest up-regulation in response to the loss of KMT2A/MLL1 (Additional file 3).

Likewise, of the genes *up*-*regulated* in response to KMT2A/MLL1 KD, a majority (448, 62%) were also up-regulated in response to Msk1 KD (Fig. 2f, lower panel). However, these overlapping, up-regulated genes represent only a minority (19%) of the 2331 genes up-regulated in total by Msk1 KD (i.e. genes whose ongoing activity is repressed by Msk1). It seems that Msk1 alone, acting either directly or indirectly through phosphorylation of regulators such as NFkB or CREB [39, 41], is frequently involved in transcriptional repression.

Ontology analysis of significant genes was consistent with KMT2A/MLL1 and Msk1 activating a wide range of gene targets (Additional file 4). Genes down-regulated by KMT2A/MLL1 *and* Msk1 KD were modestly (1.5–1.9-fold), though significantly enriched in general terms such as glycoprotein and extracellular region activity. In contrast, genes down-regulated by KD of Msk1 *alone* were enriched selectively in cell cycle terms (130 out of 543 genes), with high enrichments (3.5–9.8-fold). The gene set whose activity depends on Msk1 alone differs fundamentally from that requiring both Msk1 and KMT2A/MLL1. These very different gene sets may account for the different degrees of knockdown tolerated by the cells, almost complete for Msk1 and ~40% for KMT2A/MLL1 (Fig. 2c).

### KMT2A/MLL1 brings Msk1 to HoxA target genes to regulate their expression

**Fig. 3:**
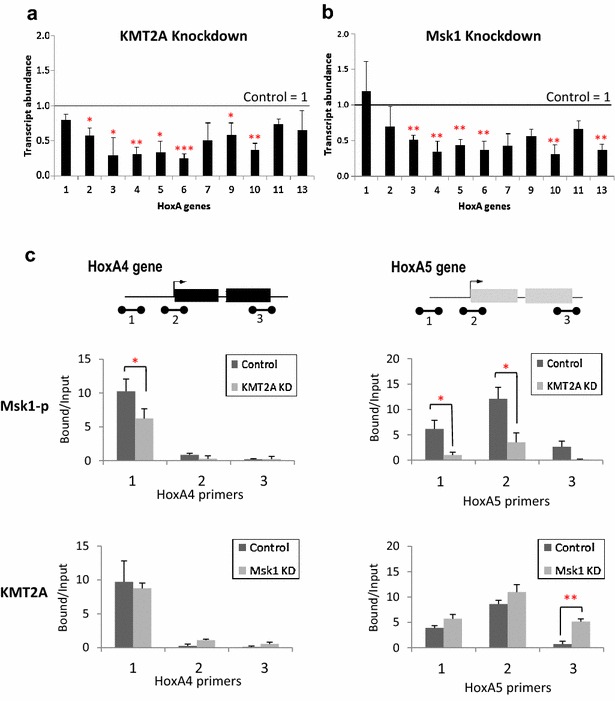
HoxA gene expression and the distribution of KMT2A/MLL1 and Msk1 change upon KMT2A/MLL1 or Msk1 knockdown. **a** RT-qPCR expression analysis of *HoxA* genes in KMT2A knockdown cells, compared to mock-transfected control cells normalised to 1.0 (*n* = 3, *T* test, *p < 0.05; **p < 0.01; ***p < 0.001). **b** RT-qPCR expression analysis of changes in expression of *HoxA* genes in Msk1 knockdown cells (*n* = 3, *T* test, *p < 0.05; **p < 0.01; ***p < 0.001). **c** Effect of KMT2A/MLL1 knockdown on Msk1 binding (*upper panels*) or Msk1 knockdown on KMT2A binding (*lower panels*) across the *HoxA4* gene (*left hand panels*) and *HoxA5* gene (*right hand panels*). Binding was normalised to mock-transfected cells (*n* = 3, *T* test, *p < 0.05; **p < 0.01; ***p < 0.001). Gene maps for *HoxA4* and *HoxA5* show exons (*boxes*), TSS (*upper arrow*) and the locations of primers 1–3 (*lower bars*)

We examined the effects of KMT2A/MLL1 and Msk1 knockdown on expression of genes within the *HoxA* cluster, important and well-described KMT2A/MLL1 target genes. Knockdown of KMT2A/MLL1 resulted in significant down-regulation of multiple *HoxA* genes, particularly those nearer the centre of the cluster (Fig. 3a). Msk1 knockdown generated a pattern of *HoxA* gene expression remarkably similar to that in KMT2A/MLL1 knockdown cells, with genes nearer the centre of the cluster, *HoxA3*–*6* and *HoxA10*, showing the greatest down-regulation (Fig. 3b).

In order to understand better the functional relationship between KMT2A/MLL1 and Msk1, we examined their interaction at the *HoxA4 and HoxA5* loci, near the centre of the cluster and amongst the most severely down-regulated genes in both knockdowns. Using ChIP-PCR with antibodies to phosphorylated Msk1, using three primer pairs spanning the gene, we found that Msk1 is normally enriched over the *HoxA4* TSS (Fig. 3c, upper left panel). This enrichment is lost when KMT2A/MLL1 is knocked down (Fig. 3c, upper left panel), suggesting that Msk1 must interact with the MLL1 complex to be recruited to these sites. In contrast, whilst a peak of KMT2A/MLL1 was detected over the *HoxA4* TSS, it did not change significantly upon Msk1 knockdown (Fig. 3c, upper right panel), indicating that Msk1 is not essential for the recruitment of the MLL1 complex. Exactly the same response to KMT2A/MLL1 and Msk1 KD was detected across the *HoxA5* locus (Fig. 3c, lower panels). It is interesting to note that the peaks of KMT2A/MLL1 and Msk1 are located at the TSS of *HoxA5*, but upstream for *HoxA4*; so, their co-location is not simple because they both are located at TSS (Fig. 3c).

**Fig. 4:**
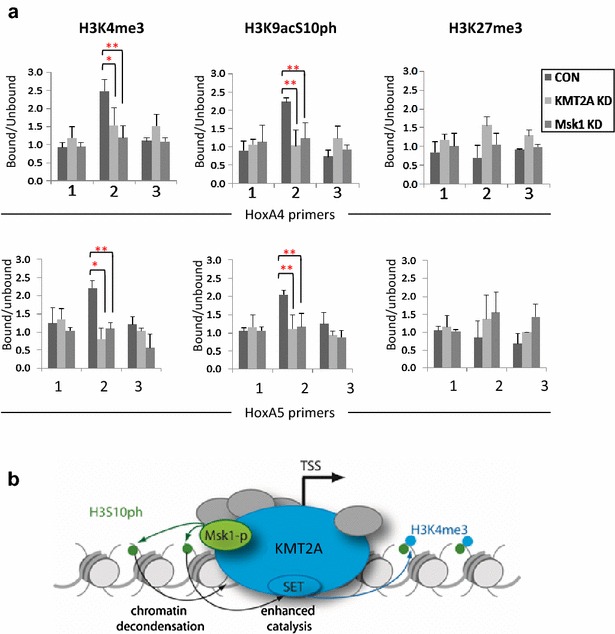
Effect of KMT2A/MLL1 or Msk1 knockdown on H3K4me3, H3K9acS10ph and H3K27me3 distribution across *HoxA4 and HoxA5.* **a** Effect of KMT2A/MLL1 or Msk1 knockdown on H3K4me3, H3K9acS10ph and H3K27me3 levels at *HoxA4* (*upper panels*) and *HoxA5* (*lower panels*). Histograms present the modification abundance at these sites as a bound/unbound ratio, where 1.0 indicates there is no enrichment (*n* = 1 or 2, *T* test, *p < 0.05; **p < 0.01). **b** Proposed model of how the KMT2A/MLL1 and Msk1 interaction within the KMT2A/MLL1 complex facilitates H3K4 methylation at transcription start sites (TSSs). Msk1-catalysed H3S10 phosphorylation directly enhances KMT2A/MLL1-catalysed H3K4 methylation and, potentially, improves access of the complex to condensed chromatin by reversing chromatin compaction

To define how KMT2A/MLL1 and Msk1 interact to regulate H3 modification levels at MLL1 complex target loci, we characterised key histone modifications at *HoxA4* and *HoxA5* in the context of KMT2A/MLL1 and Msk1 knockdown (Fig. 4a). Both H3K4me3 and H3K9acS10ph were enriched specifically at the TSS (left and middle panels). H3K4me3 fell to baseline levels, both upon KMT2A/MLL1 knockdown as expected and also in Msk1 knockdown cells (Fig. 4a, left panels). Likewise, the TSS-associated mark H3K9acS10ph was reduced both in Msk1 knockdown cells as expected and in KMT2A/MLL1 knockdown cells (Fig. 4a, middle panels). In contrast, the abundance of the silencing modification H3K27me3, used as a control, did not change upon knockdown of either protein (Fig. 4a, right panels). These results show that the KMT2A/MLL1 complex is required for recruitment of Msk1 to the *HoxA4* and *HoxA5* loci, and consistent with the proposition that Msk1-catalysed H3S10 phosphorylation facilitates the KMT2A/MLL1 complexes methyltransferase activity at these TSSs. However, the peaks of H3K4me3 and H3K9acS10ph at the *HoxA4* TSS (Fig. 4a) do not coincide with the peaks of KMT2A and Msk1 at the same locus (Fig. 3c). Thus, whilst KMT2A and Msk1 are clearly necessary for the deposition of these histone modifications, the amount of enzyme at particular locations is not the sole determinant of their levels.

## Discussion

The KMT2A/MLL1 is necessary for regulation of genes with key roles in differentiation and development [1, 2] [42–44]. It has multiple domains that mediate binding to both proteins and DNA, and allow assembly of the multi-protein complex that regulates gene expression. KMT2A/MLL1 has a SET domain that, within the complex, confers lysine methyltransferase activity specific for histone H3 lysine 4 (H3K4), and is essential for gene regulation [3, 19]. The various proteins that form, or associate with, the core MLL1 complex regulate both its ability to methylate H3K4 and its ability to locate and gain access to its target genes, but the mechanisms that underpin these processes are not yet understood [1, 7]. Here, we show that the kinase Msk1, part of the MAPK signalling pathway, physically interacts with the MLL1 complex, is essential for the regulation of KMT2A/MLL1 target genes and, at *HoxA4* and *HoxA5*, that this is via regulation of H3K4 methylation.

Four lines of evidence demonstrate the physical association of Msk1 with the KMT2A/MLL1 complex. First, KMT2A/MLL1 and Msk1 co-immunoprecipitate from extracts of several different cell types. Second, the two proteins co-localise across *HoxA* genes (known targets). Third, knockdown of Msk1 diminishes methylation of H3K4 at MLL1 complex target sites. Likewise, KMT2A/MLL1 knockdown diminishes H3S10 phosphorylation at the same sites, suggesting that, in the absence of the KMT2A/MLL1 complex, Msk1 is not appropriately targeted. This was confirmed by showing that KMT2A/MLL1 knockdown prevented Msk1 recruitment to *HoxA4* and *HoxA5* genes, whilst Msk1 knockdown did not disrupt KMT2A/MLL1 binding. Finally, our finding that expression of 81% of KMT2A/MLL1-regulated genes are influenced by Msk1 knockdown, shows that the functional interaction between Msk1 and the KMT2A/MLL1 complex in vivo is very frequent. Because our knockdown experiments targeting Msk1 left its close homologue Msk2 unaffected, we conclude that the effects we observe are mediated by Msk1 alone.

Msk1 is known to phosphorylate histone H3 at serines 10 and 28, as well as key regulatory proteins such as cAMP response element binding (CREB) [41] and NFκB [39]. All of these substrates can, potentially, alter gene expression. The choice of which substrate, or even which lysine, to phosphorylate is likely to be context dependent [45, 46]. The catalytic properties of Msk1, previous data and the present results suggest that the enzyme regulates the KMT2A/MLL1 complex in one or both of two general ways, namely by enhancing SET domain-catalysed H3K4 methylation and/or by allowing the complex to access condensed chromatin. We have shown previously that SET domain methyltransferase activity is enhanced by the acetylation and phosphorylation of the H3 substrate at lysines 9 and 14 and serine 10, respectively [33]. The most effective catalytic improvement was provided by the fully modified substrate (H3K9acS10phK14ac) [33]. We have now shown that recruitment of Msk1 to KMT2A/MLL1 responsive genes and consequent increases in H3S10 phosphorylation leads to increased H3K4 methylation and up-regulation of transcription. In light of these data, we propose that (in addition to its other activities) Msk1-mediated phosphorylation of H3S10 directly stimulates the ability of the KMT2A/MLL1 SET domain to methylate H3K4 in vivo. It may be that the recruitment of the lysine acetyltransferase CBP by the KMT2A/MLL1 complex [31] also contributes to enhancement of H3K4 methylation through enhanced acetylation of H3K9 and K14. This model is shown in Fig. 4b.

H3 serines 10 and 28 are both adjacent to lysine residues whose various modifications contribute to long- and short-term control of gene expression. H3K9 acetylation is associated with active gene promoters [47] whilst its methylation is associated with silent, condensed chromatin (including centric heterochromatin) [48]. H3K9me-mediated chromatin condensation is brought about largely through selective binding of heterochromatin protein 1 (HP1) [49, 50]. Msk1 phosphorylation of H3S10 appears to act in this process as a ‘phospho-switch’ that can block HP1 binding to H3K9me3, and even displace pre-bound HP1 [51, 52], thereby preventing or reversing gene silencing. This has been characterised on the mouse mammary tumour virus (MMTV) promoter, where Msk1 recruitment, along with the progesterone receptor (PR) and the extracellular signal-regulated kinase (Erk), leads to phosphorylation of H3S10 at the promoter, displacement of HP1 and recruitment of RNA polymerase II and the activator protein Brg1 [53]. We propose that Msk1 facilitates binding of the KMT2A/MLL1 complex to some target genes by H3S10 phosphorylation and consequent diminution of HP1-mediated chromatin condensation (Fig. 4b).

Methylation of H3K27 is necessary for gene silencing by the Polycomb repression complex PRC2 [54] and is put in place, and bound, by a component of the complex, the methyltransferase Ezh2 [55]. Genes can be protected against PRC2-mediated silencing by H3K27 acetylation [56], and by modification of other H3 lysines [46, 57–59], but phosphorylation of H3 at serine 28, catalysed by MSK1/2, is known to displace PRC2, presumably by disrupting binding to the adjacent, methylated residue [60, 61]. This is consistent with the increase in H3K28ph abundance observed at the promoter regions of genes that are transcriptionally activated when quiescent 3T3 cells are stimulated back into growth [62]. H3S28 phosphorylation allows Polycomb-silenced genes to be reactivated without the need to demethylate H3K27 and may play a role in allowing the KMT2A/MLL1 complex to access such genes.

In light of these results, the association of Msk1 with the MLL1 complex confers the potential ability to reverse both HP1-mediated and Polycomb-mediated chromatin silencing, as a prelude to selective gene activation. Whatever the detailed mechanisms may prove to be, the close physical and functional association between the KMT2A/MLL1 complex and Msk1 indicates novel routes by which the complex can implement its role as a transcriptional activator and developmental regulator.

## Conclusion

The histone kinase Msk1 physically associates in vivo with the KMT2A/MLL1 complex in human and mouse cells and is essential for the regulation of key developmental target genes. This represents a direct functional link between the MAPK cell signalling pathway and KMT2A-/MLL1-mediated gene regulation. At least, part of this regulatory role is accomplished through histone modification, specifically methylation of H3K4 and phosphorylation of H3S10 and possibly H3S28.

## Methods

### Cell culture

Human lymphoblastoid cells (LCLs) with a normal karyotype were generated by M. Rowe (Uni. Birmingham) and grown in RPMI 1640, 100 µg/ml streptomycin, 100 U/ml penicillin and 10% FBS (Foetal bovine serum, Invitrogen) at 37 °C, 5% CO_2_. Human embryonic kidney cells (HEKs) were cultured in DMEM, 100 µg/ml streptomycin, 100 U/ml penicillin and 10% FBS at 37 °C, 5% CO_2_. Mouse embryonic fibroblasts (MEFs) were generated in-house, via isolation from Balb/c wild-type mouse embryos between day 12 and 15 of gestation, and cultured in DMEM, 100 µg/ml streptomycin, 100 U/ml penicillin, 1/100 MEM non-essential amino acids, 1/1000 2-mercaptoethanol and 10% FBS at 37 °C, 5% CO_2_. Only cells between passage 2 and 4 were used. *Drosophila* SL2 (Schneider line-2) cells were cultured in Schneider’s medium supplemented with 8% FBS and 1/100 penicillin/streptomycin, and incubated at 26 °C.

### RNA extraction and RT-qPCR

RNA was isolated using RNeasy Minikit (Quaigen), and reverse transcription/cDNA amplification performed in one step using QuantiTech™ Sybr^®^ Green Mix (Qiagen), according to the manufacturer’s instructions. Reactions used commercial primers (Qiagen: Msk1 QT00141554, actin QT01336772, HoxA1 QT00248322, HoxA2 QT0062812, HoxA3 QT00138264, HoxA4 QT00174986, HoxA5 QT00098819, HoxA6 QT00140742, HoxA7 QT00168707, HoxA9 QT00108885, HoxA10 QT00240212, HoxA11 QT00250404, HoxA13 QT0027642, MLL1 QT00240954, MLL2 QT01075620, MLL3 QT00310527, MLL5 QT00279426), with three analyses performed for each sample. RT-qPCR was performed on a 7900 HT machine (Applied Biosystems).

### Isolation of proteins and detection

Cells were washed and incubated in lysis buffer [20 mM Tris–HCl pH 7.4, 100 mM EDTA, 10 mM NaCl, 1% Triton X-100, 1 mM β-glycerophosphate, 1 mM EGTA, 5 mM sodium pyrophosphate, ‘Complete’ protease inhibitor (*Roche*)] for 30 min on ice. Insoluble material was removed by centrifugation and protein concentration determined by *Coomassie Plus* protein assay (Thermo Scientific). Proteins were separated by gel electrophoresis and stained with silver nitrate or blotted for western analysis. Primary antibody binding was detected by fluorescently tagged secondary antibodies (Licor) and gel loading normalised using anti-actin or anti-Pol II antibodies (Abcam).

### Antibodies

Antibody for H3K4me3 (R612) was generated in-house [63, 64]. Other antibodies were obtained commercially: H3K9acS10p [Abcam ab12181]; H3K27me3 [Millipore 07-449]; phosphorylated Msk1, this antibody is specific for Msk1 phosphorylated at S360, one of the last residues to become phosphorylated and detects active Msk1 [Abcam ab81294]; KMT2A [Millipore 05-764]; Msk2 [R&D systems MAB2310]; actin [Abcam ab1801]; RNA Pol II [Abcam ab5408]; NFκB [Abcam ab7970-1]; FLAG [Sigma F1804].

### Chromatin immunoprecipitation (ChIP)

Immunoprecipitation of micrococcal nuclease-digested, native chromatin coupled to quantitative PCR (qPCR) was used to determine levels of histone modification at selected genomic regions, as previously described [65, 66].

Immunoprecipitation of formaldehyde cross-linked chromatin (X-ChIP) in MEFs was carried out essentially as described [67, 68]. Briefly, MEFs were cross-linked with 1% para-formaldehyde in medium for 10 min at RT. The reaction was quenched by the addition of 200 mM glycine before washing the cells twice with ice-cold PBS. Cells were resuspended in lysis buffer [1% SDS, 10 mM EDTA, 50 mM Tris–HCl, pH 8, complete inhibitor cocktail(Roche)] to a concentration of 10^5^ cells in 500 μl and sonicated at high power with 30 s ON/OFF for 7 cycles (Bioruptor, Diagenode). The chromatin was diluted 1:2 with dilution buffer [1% Triton X-100, 2 mM EDTA, 20 mM Tris–HCl, pH 8, 150 mM NaCl, complete inhibitor cocktail (Roche)]. Protein G Dynabeads (Thermo Fischer) were prepared resuspended in citrate–phosphate buffer (0.1 M citric acid, 0.1 M Na_2_HPO_4_) with 0.5% BSA. Beads were coated with antibodies for 2 h at 4 °C. The beads were washed twice, added to the chromatin solution and incubated for 4 h at 4 °C. The beads were washed twice with low-salt buffer (1% Triton X-100, 0.1% SDS, 2 mM EDTA, 150 mM NaCl, 20 mM Tris–HCl, pH 8) and twice with high-salt buffer (1% Triton X-100, 0.1% SDS, 2 mM EDTA, 100 mM NaCl, 20 mM Tris–HCl, pH 8). The bound material was eluted with Elution buffer (100 mM NaHCO_3_, 1% SDS decross-linked, and the DNA extracted with QIAquick PCR purification kit (QIAGEN). Primer pairs used for analysing ChIP DNA by qPCR are listed in Additional file 5.

### Co-immunoprecipitation

Co-immunoprecipitation experiments were carried out as described before, using 1 × 10^8^ cells per precipitation and in the presence of 50 µg/ml ethidium bromide, in order to release chromatin bound proteins from DNA [69–72]. Cells were lysed in NP40 buffer [1% NP40, 10% glycerol, 50 mM Tris pH 7.5, 0.1% sodium azide, 150 mM NaCl, *complete* proteinase inhibitor (Roche)] for 20 min on ice before sonication (high power, 10 s, Bioruptor Diagenode). The lysate was cleared by centrifugation (10 min, 17000*g*, 4 °C) and pre-cleaned by adding protein A Sepharose beads for 30 min at 4 °C. The cleaned lysate was incubated overnight with 15 µg of antibody at 4 °C on a rotating wheel and then with 40 µl beads for 3 h at RT. The beads were washed three times with high-salt buffer (1% NP40, 10% glycerol, 50 mM Tris pH 7.5, 0.1% sodium azide, 200 mM NaCl). The beads were analysed on a SDS gel and western analysis, together with a no-antibody control and 10% input samples.

### Overexpression of FLAG-MLL1 and FLAG precipitation

HEK293 cells were split the day before transfection and 4 × 10^6^ cells seeded into 10 cm plates in complete medium. The next day, a mix of 15 μg vector DNA (pMSCV-FLAG-MLL1, R. Slany, University Erlangen), 1000 μl OptiMEM (Gibco) and 60 μl of polyethyleneimine (1 mg/ml, Polyscience Inc.) per plate was incubated for 15 min at RT prior to addition to the plates. The cells were cultured at 37 °C, 5% CO_2_ for 3 days and harvested, washed with TBS and resuspended in lysis buffer (1× TBS, 1 mM EDTA, 1% TritonX-100). Cells were incubated on ice for 30 min prior to centrifuging (15 min, 13,000 rpm, 4 °C), addition of 50 μl anti-FLAG M2 magnetic beads (Sigma) and incubation for 2 h at RT with rotation. Beads were washed with TBS and FLAG-MLL1 was eluted using 5 μg of 3× FLAG peptide (Sigma) and diluted in 500 μl TBS. The beads were incubated twice with 250 μl elution for 30 min, 4 °C with rotation and the supernatants pooled.

### shRNA knockdowns

shRNA vectors were purchased from Origene (Msk1: TR504795; MLL1: TR517798) and transfected into MEFs by electroporation (GenePulser XCell, Bio-Rad). MEFs were brought into a single cell suspension by trypsination and washed with ice-cold PBS. 4 × 10^6^ cells per transfection were resuspended in 400 μl electroporation buffer (10 mM Hepes, pH 7.5, 135 mM KCl, 2 mM MgCl_2_, 5 mM EGTA, 25% FBS) and transferred to a 4 mm electroporation cuvette (Bio-Rad). 20 μg of vector DNA was added to the cells immediately before transfection and incubated on ice for 1 min. Cells were transfected with one pulse (300 V, 600 μF, 1000 Ω) and allowed to recover (RT, 1 min), before addition of 10 ml pre-warmed MEF medium and incubation at 37 °C, 5% CO_2_ for 6 h. Non-transfected cells were removed by puromycin treatment (10 μg/ml, 14 h). After 20 h, cells were harvested. The KD efficiency is determined by quantitation of Wester blots using the corresponding loading controls for normalisation.

### Gene expression arrays

Expression analysis was performed on MEFs transfected with knockdown shRNA vectors, using NimbleGen arrays (100718_MM9_EXP_HX12) containing 12 individual 135 k arrays with 3 probes per sequence for 42,576 sequences. 10 μg of RNA per array was isolated (RNeasy, Qiagen) and reverse transcribed using the cDNA synthesis system (NimbleGen). cDNA samples were labelled with the NimbleGen One-Colour DNA labelling kit and subsequently hybridised on the array, washed and scanned with the MS 200 Microarray Scanner (NimbleGen). Four replicate transfections were carried out for each knockdown and control but one control and one MSK1 KD replicate were eliminated as outliers. Data were extracted in DEVA (Nimblegen), processed with R (background correction and normalisation) and analysed with MeV (clustering, statistical analysis and visualisation). Statistical significance of co-regulated genes was analysed by 2 × 2 contingency tables and Chi-squared analysis with Yates correction (GraphPad, www.graphpad.com). Microarray data have been deposited in GEO (www.ncbi.nlm.nih.gov/geo), accession number GSE89141.

## Additional files

**Additional file 1.** Co-immunoprecipitation of KMT2A and Msk1. The complete membranes from the Western blot images shown in Fig. 1a, b.

**Additional file 2.** Validation of microarray data. Primer pairs for randomly selected genes that were up- (Hemt1/Hsph1/Ifnar2/Tet3) or down-regulated 9Il1b/Ly9/Ttk/Kif20b) according to the microarray analysis were designed. The genes were tested with RT-qPCR on KMT2A/MLL1 knockdown or Msk1 knockdown RNA (n = 3).

**Additional file 3.** Knockdown of KMT2A/MLL1 does not diminish Msk1, nor does Msk1 KD diminish KMT2A. Changes in KMT2A/MLL1 and Msk1 transcript abundance in KMT2A/MLL1 and Msk1 knockdown cells. Transcript levels are presented as fold change and normalised to the mock-transfected controls (n = 3/4, T-test p < 0.05*, p < 0.01**, p < 0.001***).

**Additional file 4.** Ontology analysis of genes affected by KMT2A1 and/or Msk1 knockdown.

**Additional file 5.** qPCR primer sets for C-ChIP and X-ChIP analysis.

## Abbreviations

ASH2L: absent, small or homeotic 2-like
H3: histone H3
KD: knockdown
KMT: lysine methyltransferase
MLL: mixed-lineage leukaemia
Msk: nuclear mitogen- and stress-activated protein kinase
NFκB: nuclear factor κB
RbBP5: retinoblastoma-binding protein 5
RT: room temperature
SET domain: suppressor of variegation, enhancer of zeste, trithorax domain
SOTA: self-organising tree algorithm
TSS: transcription start site
WDR: WD-40 repeat 5

## References

[1] Rao RC, Dou Y (2015). Hijacked in cancer: the KMT2 (MLL) family of methyltransferases. Nat Rev Cancer.

[2] Schuettengruber B (2011). Trithorax group proteins: switching genes on and keeping them active. Nat Rev Mol Cell Biol.

[3] Milne TA (2002). MLL targets SET domain methyltransferase activity to Hox gene promoters. Mol Cell.

[4] Cui K (2009). Chromatin signatures in multipotent human hematopoietic stem cells indicate the fate of bivalent genes during differentiation. Cell Stem Cell.

[5] Krivtsov AV, Armstrong SA (2007). MLL translocations, histone modifications and leukaemia stem-cell development. Nat Rev Cancer.

[6] Kandoth C (2013). Mutational landscape and significance across 12 major cancer types. Nature.

[7] van Nuland R (2013). Quantitative dissection and stoichiometry determination of the human SET1/MLL histone methyltransferase complexes. Mol Cell Biol.

[8] Patel A (2009). On the mechanism of multiple lysine methylation by the human mixed lineage leukemia protein-1 (MLL1) core complex. J Biol Chem.

[9] Dou Y (2006). Regulation of MLL1 H3K4 methyltransferase activity by its core components. Nat Struct Mol Biol.

[10] Hughes CM (2004). Menin associates with a trithorax family histone methyltransferase complex and with the hoxc8 locus. Mol Cell.

[11] Yokoyama A, Cleary ML (2008). Menin critically links MLL proteins with LEDGF on cancer-associated target genes. Cancer Cell.

[12] Mo R, Rao SM, Zhu YJ (2006). Identification of the MLL2 complex as a coactivator for estrogen receptor alpha. J Biol Chem.

[13] Tyagi S (2007). E2F activation of S phase promoters via association with HCF-1 and the MLL family of histone H3K4 methyltransferases. Mol Cell.

[14] Lee J (2009). A tumor suppressive coactivator complex of p53 containing ASC-2 and histone H3-lysine-4 methyltransferase MLL3 or its paralogue MLL4. Proc Natl Acad Sci USA.

[15] Patel A (2008). A conserved arginine-containing motif crucial for the assembly and enzymatic activity of the mixed lineage leukemia protein-1 core complex. J Biol Chem.

[16] Wu L (2013). ASH2L regulates ubiquitylation signaling to MLL: trans-regulation of H3 K4 methylation in higher eukaryotes. Mol Cell.

[17] Buecker C, Wysocka J (2012). Enhancers as information integration hubs in development: lessons from genomics. Trends Genet.

[18] Calo E, Wysocka J (2013). Modification of enhancer chromatin: what, how, and why?. Mol Cell.

[19] Ruthenburg AJ, Allis CD, Wysocka J (2007). Methylation of lysine 4 on histone H3: intricacy of writing and reading a single epigenetic mark. Mol Cell.

[20] Ruthenburg AJ (2007). Multivalent engagement of chromatin modifications by linked binding modules. Nat Rev Mol Cell Biol.

[21] Nayak A (2014). The SUMO-specific isopeptidase SENP3 regulates MLL1/MLL2 methyltransferase complexes and controls osteogenic differentiation. Mol Cell.

[22] Hsieh JJ, Cheng EH, Korsmeyer SJ (2003). Taspase1: a threonine aspartase required for cleavage of MLL and proper HOX gene expression. Cell.

[23] Hsieh JJ (2003). Proteolytic cleavage of MLL generates a complex of N- and C-terminal fragments that confers protein stability and subnuclear localization. Mol Cell Biol.

[24] Yokoyama A (2002). Leukemia proto-oncoprotein MLL is proteolytically processed into 2 fragments with opposite transcriptional properties. Blood.

[25] Yokoyama A (2013). MLL becomes functional through intra-molecular interaction not by proteolytic processing. PLoS ONE.

[26] Daser A, Rabbitts TH (2004). Extending the repertoire of the mixed-lineage leukemia gene MLL in leukemogenesis. Genes Dev.

[27] Zeleznik-Le NJ, Harden AM, Rowley JD (1994). 11q23 translocations split the “AT-hook” cruciform DNA-binding region and the transcriptional repression domain from the activation domain of the mixed-lineage leukemia (MLL) gene. Proc Natl Acad Sci USA.

[28] Birke M (2002). The MT domain of the proto-oncoprotein MLL binds to CpG-containing DNA and discriminates against methylation. Nucleic Acids Res.

[29] Nakamura T (2002). ALL-1 is a histone methyltransferase that assembles a supercomplex of proteins involved in transcriptional regulation. Mol Cell.

[30] Ernst P (2001). MLL and CREB bind cooperatively to the nuclear coactivator CREB-binding protein. Mol Cell Biol.

[31] Arai M, Dyson HJ, Wright PE (2010). Leu628 of the KIX domain of CBP is a key residue for the interaction with the MLL transactivation domain. FEBS Lett.

[32] Dou Y (2005). Physical association and coordinate function of the H3 K4 methyltransferase MLL1 and the H4 K16 acetyltransferase MOF. Cell.

[33] Nightingale KP (2007). Cross-talk between histone modifications in response to histone deacetylase inhibitors: MLL4 links histone H3 acetylation and histone H3K4 methylation. J Biol Chem.

[34] Boudadi E (2013). The histone deacetylase inhibitor sodium valproate causes limited transcriptional change in mouse embryonic stem cells but selectively overrides Polycomb-mediated Hoxb silencing. Epigenet Chromatin.

[35] Dyson MH (2005). MAP kinase-mediated phosphorylation of distinct pools of histone H3 at S10 or S28 via mitogen- and stress-activated kinase 1/2. J Cell Sci.

[36] Soloaga A (2003). MSK2 and MSK1 mediate the mitogen- and stress-induced phosphorylation of histone H3 and HMG-14. EMBO J.

[37] McCoy CE (2005). MSK1 activity is controlled by multiple phosphorylation sites. Biochem J.

[38] Arthur JS (2008). MSK activation and physiological roles. Front Biosci.

[39] Vermeulen L (2003). Transcriptional activation of the NF-kappaB p65 subunit by mitogen- and stress-activated protein kinase-1 (MSK1). EMBO J.

[40] Guenther MG (2005). Global and Hox-specific roles for the MLL1 methyltransferase. Proc Natl Acad Sci USA.

[41] Deak M (1998). Mitogen- and stress-activated protein kinase-1 (MSK1) is directly activated by MAPK and SAPK2/p38, and may mediate activation of CREB. EMBO J.

[42] Hess JL (1997). Defects in yolk sac hematopoiesis in Mll-null embryos. Blood.

[43] Terranova R (2006). Histone and DNA methylation defects at Hox genes in mice expressing a SET domain-truncated form of Mll. Proc Natl Acad Sci USA.

[44] Jude CD (2007). Unique and independent roles for MLL in adult hematopoietic stem cells and progenitors. Cell Stem Cell.

[45] Drobic B (2010). Promoter chromatin remodeling of immediate-early genes is mediated through H3 phosphorylation at either serine 28 or 10 by the MSK1 multi-protein complex. Nucleic Acids Res.

[46] Sabbattini P (2014). An H3K9/S10 methyl-phospho switch modulates Polycomb and Pol II binding at repressed genes during differentiation. Mol Biol Cell.

[47] Karmodiya K (2012). H3K9 and H3K14 acetylation co-occur at many gene regulatory elements, while H3K14ac marks a subset of inactive inducible promoters in mouse embryonic stem cells. BMC Genom.

[48] Barski A (2007). High-resolution profiling of histone methylations in the human genome. Cell.

[49] Bannister AJ (2001). Selective recognition of methylated lysine 9 on histone H3 by the HP1 chromo domain. Nature.

[50] Eskeland R, Eberharter A, Imhof A (2007). HP1 binding to chromatin methylated at H3K9 is enhanced by auxiliary factors. Mol Cell Biol.

[51] Fischle W (2005). Regulation of HP1-chromatin binding by histone H3 methylation and phosphorylation. Nature.

[52] Mateescu B (2004). Tethering of HP1 proteins to chromatin is relieved by phosphoacetylation of histone H3. EMBO Rep.

[53] Vicent GP (2006). Induction of progesterone target genes requires activation of Erk and Msk kinases and phosphorylation of histone H3. Mol Cell.

[54] Margueron R, Reinberg D (2011). The Polycomb complex PRC2 and its mark in life. Nature.

[55] Kuzmichev A (2002). Histone methyltransferase activity associated with a human multiprotein complex containing the Enhancer of zeste protein. Genes Dev.

[56] Tie F (2009). CBP-mediated acetylation of histone H3 lysine 27 antagonizes Drosophila Polycomb silencing. Development.

[57] Yuan W (2011). H3K36 methylation antagonizes PRC2-mediated H3K27 methylation. J Biol Chem.

[58] Schmitges FW (2011). Histone methylation by PRC2 is inhibited by active chromatin marks. Mol Cell.

[59] Xu C (2010). Binding of different histone marks differentially regulates the activity and specificity of polycomb repressive complex 2 (PRC2). Proc Natl Acad Sci USA.

[60] Gehani SS (2010). Polycomb group protein displacement and gene activation through MSK-dependent H3K27me3S28 phosphorylation. Mol Cell.

[61] Lau PN, Cheung P (2011). Histone code pathway involving H3 S28 phosphorylation and K27 acetylation activates transcription and antagonizes polycomb silencing. Proc Natl Acad Sci USA.

[62] Sawicka A (2014). H3S28 phosphorylation is a hallmark of the transcriptional response to cellular stress. Genome Res.

[63] Terrenoire E (2010). Immunostaining of modified histones defines high-level features of the human metaphase epigenome. Genome Biol.

[64] White DA, Belyaev ND, Turner BM (1999). Preparation of site-specific antibodies to acetylated histones. Methods.

[65] Halsall JA (2015). Cells adapt to the epigenomic disruption caused by histone deacetylase inhibitors through a coordinated, chromatin-mediated transcriptional response. Epigenet Chromatin.

[66] O’Neill LP, Turner BM (2003). Immunoprecipitation of native chromatin: NChIP. Methods.

[67] Breiling A, Orlando V. Binding sights in chromatin by X-ChIP. In: Mapping protein/DNA interactions by cross-linking. Paris: Institut national de la sante et de la recherche medicale (INSERM); 2001. 21413365

[68] Kuo MH, Allis CD (1999). In vivo cross-linking and immunoprecipitation for studying dynamic Protein:DNA associations in a chromatin environment. Methods.

[69] Bonifacino, JS, Dell’Angelica EC, Springer TA. Immunoprecipitation. Curr Protoc Immunol. 2001;Chapter 8:Unit 8.3. doi:10.1002/0471142735.im0803s41. 10.1002/0471142735.im0803s41 18432858

[70] Harlow E, Lane D. Immunoprecipitation: purifying the immune complexes. CSH Protoc. 2006;2006(4). doi:10.1101/pdb.prot4536. 10.1101/pdb.prot4536 22485918

[71] Schroter H (1985). DNA intercalators induce specific release of HMG 14, HMG 17 and other DNA-binding proteins from chicken erythrocyte chromatin. EMBO J.

[72] Lai JS, Herr W (1992). Ethidium bromide provides a simple tool for identifying genuine DNA-independent protein associations. Proc Natl Acad Sci USA.

