# Supplementary material for: Protein kinase Msk1 physically and functionally interacts with the KMT2A/MLL1 methyltransferase complex and contributes to the regulation of multiple target genes
Source: Epigenetics Chromatin. 2016 Nov 11;9:52. doi: 10.1186/s13072-016-0103-3 (PMC5106815; doi:10.1186/s13072-016-0103-3)
Supplement: Supplementary file 1 — Additional file 1. Co-immunoprecipitation of KMT2A and Msk1. The complete membranes from the Western blot images shown in Fig. 1a, b. [file 13072_2016_103_MOESM1_ESM.pptx]

## Slide 1
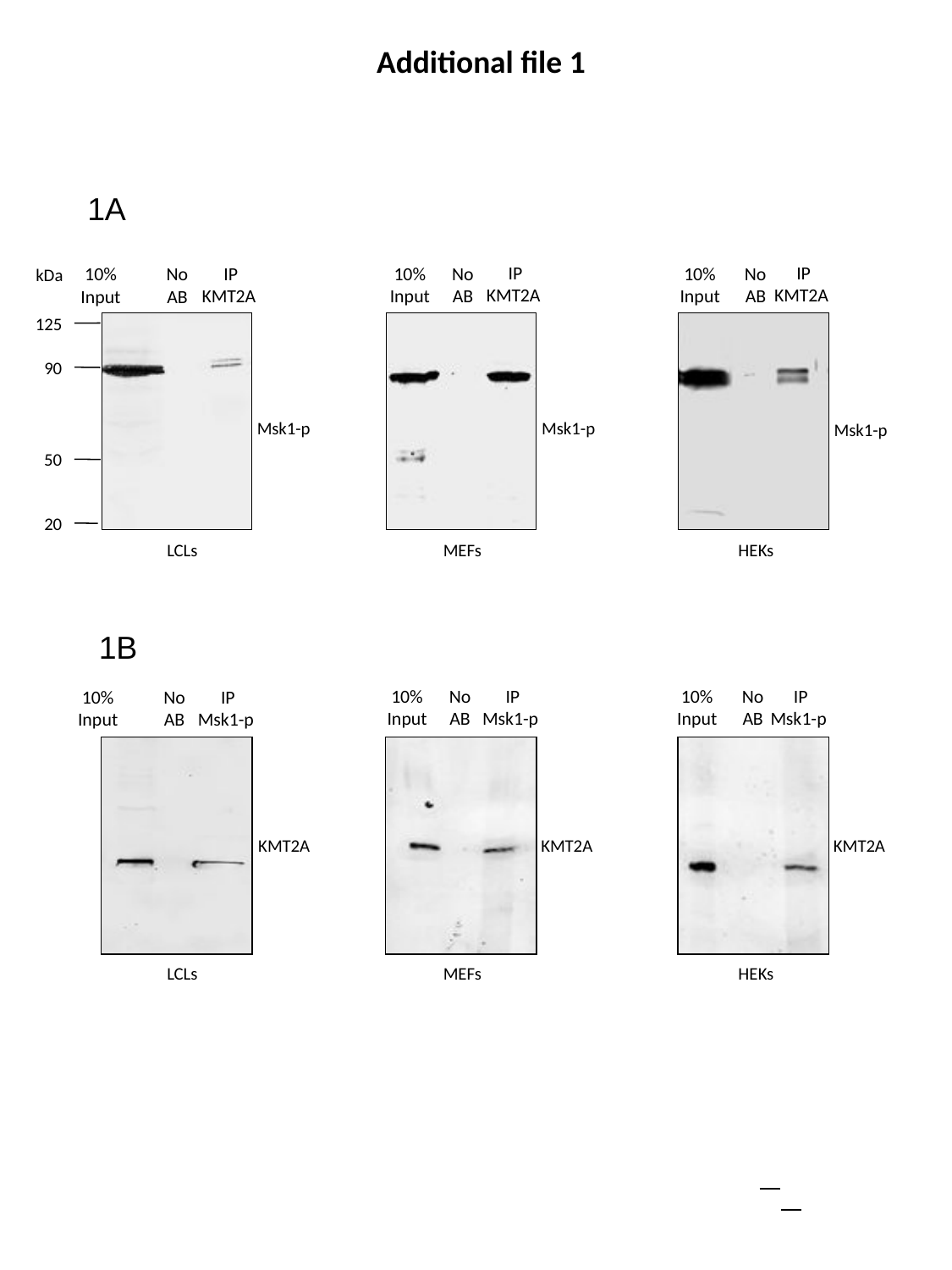

Additional file 1
1A
IP
KMT2A
IP
KMT2A
10%
Input
No
AB
10%
Input
No
AB
IP
KMT2A
10%
Input
No
AB
kDa
125
90
Msk1-p
Msk1-p
Msk1-p
50
20
HEKs
LCLs
MEFs
1B
IP
Msk1-p
IP
Msk1-p
10%
Input
No
AB
10%
Input
No
AB
IP
Msk1-p
10%
Input
No
AB
KMT2A
KMT2A
KMT2A
HEKs
LCLs
MEFs
