# Supplementary material for: Protein kinase Msk1 physically and functionally interacts with the KMT2A/MLL1 methyltransferase complex and contributes to the regulation of multiple target genes
Source: Epigenetics Chromatin. 2016 Nov 11;9:52. doi: 10.1186/s13072-016-0103-3 (PMC5106815; doi:10.1186/s13072-016-0103-3)
Supplement: Supplementary file 2 — Additional file 2. Validation of microarray data. Primer pairs for randomly selected genes that were up- (Hemt1/Hsph1/Ifnar2/Tet3) or down-regulated 9Il1b/Ly9/Ttk/Kif20b) according to the microarray analysis were designed. The genes were tested with RT-qPCR on KMT2A/MLL1 knockdown or Msk1 knockdown RNA (n = 3). [file 13072_2016_103_MOESM2_ESM.pptx]

## Slide 1
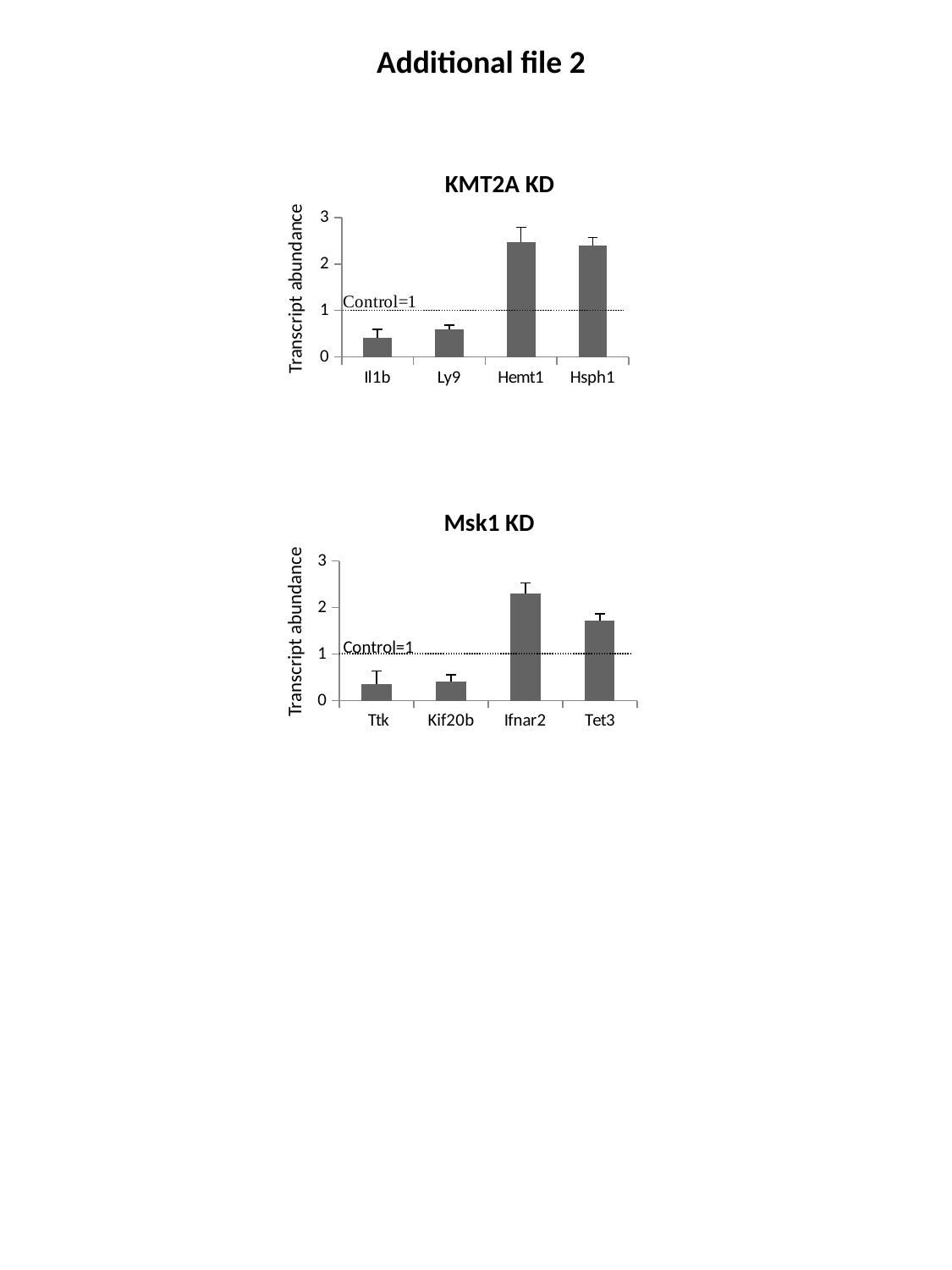

Additional file 2
### Chart
| Category | |
|---|---|
| Il1b | 0.4197039102877536 |
| Ly9 | 0.5990771711986086 |
| Hemt1 | 2.466261750577036 |
| Hsph1 | 2.389230700305189 |KMT2A KD
Transcript abundance
### Chart
| Category | 0.348712739 0.412394755 2.302384113 1.714807126 |
|---|---|
| Ttk | 0.34871273913020695 |
| Kif20b | 0.4123947550067851 |
| Ifnar2 | 2.3023841127652975 |
| Tet3 | 1.7148071255584074 |Msk1 KD
Transcript abundance
Control=1
