# Supplementary material for: Protein kinase Msk1 physically and functionally interacts with the KMT2A/MLL1 methyltransferase complex and contributes to the regulation of multiple target genes
Source: Epigenetics Chromatin. 2016 Nov 11;9:52. doi: 10.1186/s13072-016-0103-3 (PMC5106815; doi:10.1186/s13072-016-0103-3)
Supplement: Supplementary file 3 — Additional file 3. Knockdown of KMT2A/MLL1 does not diminish Msk1, nor does Msk1 KD diminish KMT2A. Changes in KMT2A/MLL1 and Msk1 transcript abundance in KMT2A/MLL1 and Msk1 knockdown cells. Transcript levels are presented as fold change and normalised to the mock-transfected controls (n = 3/4, T-test p < 0.05*, p < 0.01**, p < 0.001***). [file 13072_2016_103_MOESM3_ESM.pptx]

## Slide 1
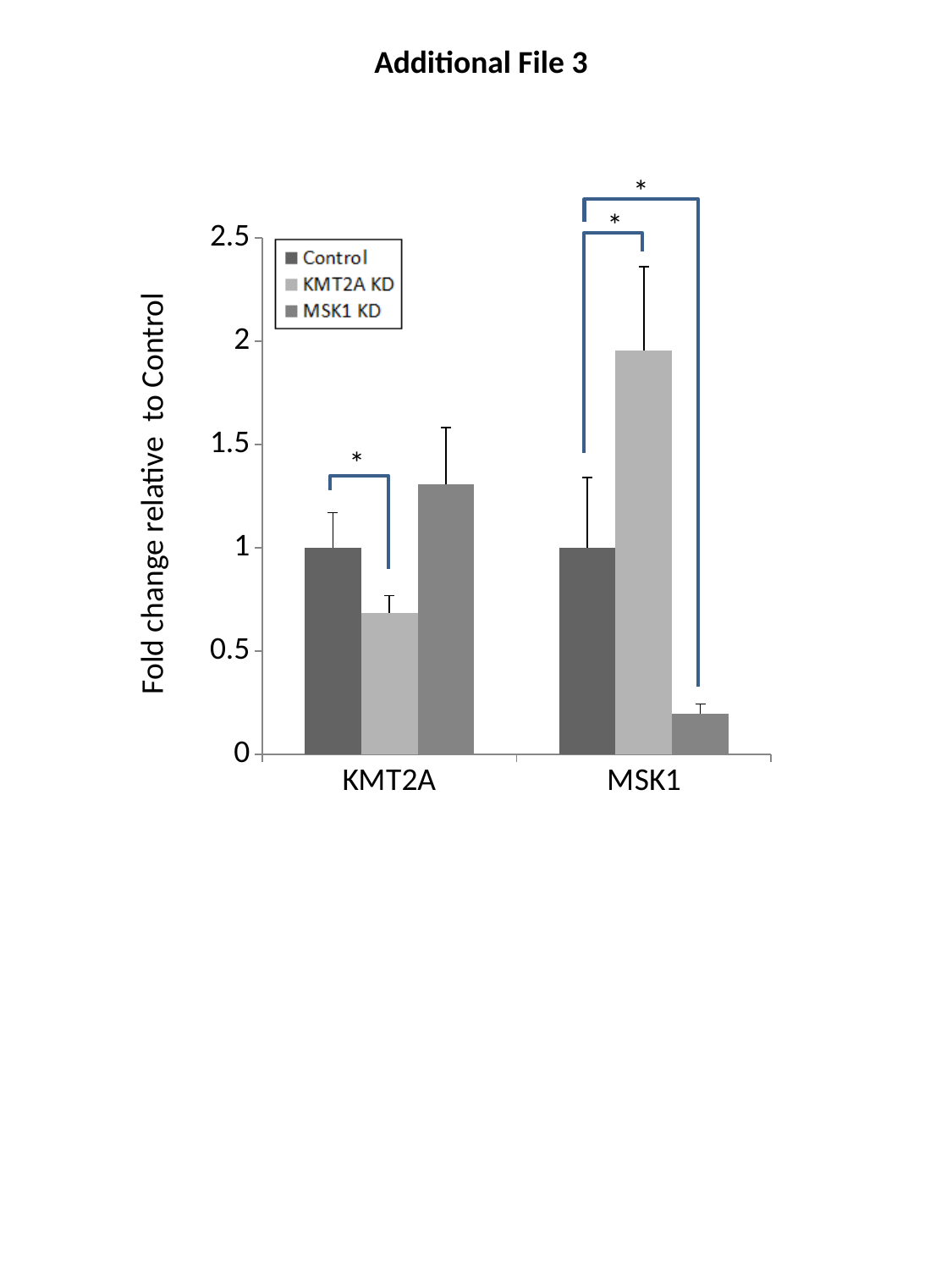

Additional File 3
*
*
### Chart
| Category | Control | KMT2A KD | MSK1 KD |
|---|---|---|---|
| KMT2A | 1.0 | 0.6835332100629367 | 1.3076465467183922 |
| MSK1 | 1.0 | 1.9561395375395934 | 0.19377968354932323 |
*
Fold change relative to Control
