# Supplementary material for: Protein kinase Msk1 physically and functionally interacts with the KMT2A/MLL1 methyltransferase complex and contributes to the regulation of multiple target genes
Source: Epigenetics Chromatin. 2016 Nov 11;9:52. doi: 10.1186/s13072-016-0103-3 (PMC5106815; doi:10.1186/s13072-016-0103-3)
Supplement: Supplementary file 4 — Additional file 4. Ontology analysis of genes affected by KMT2A1 and/or Msk1 knockdown. [file 13072_2016_103_MOESM4_ESM.pptx]

## Slide 1
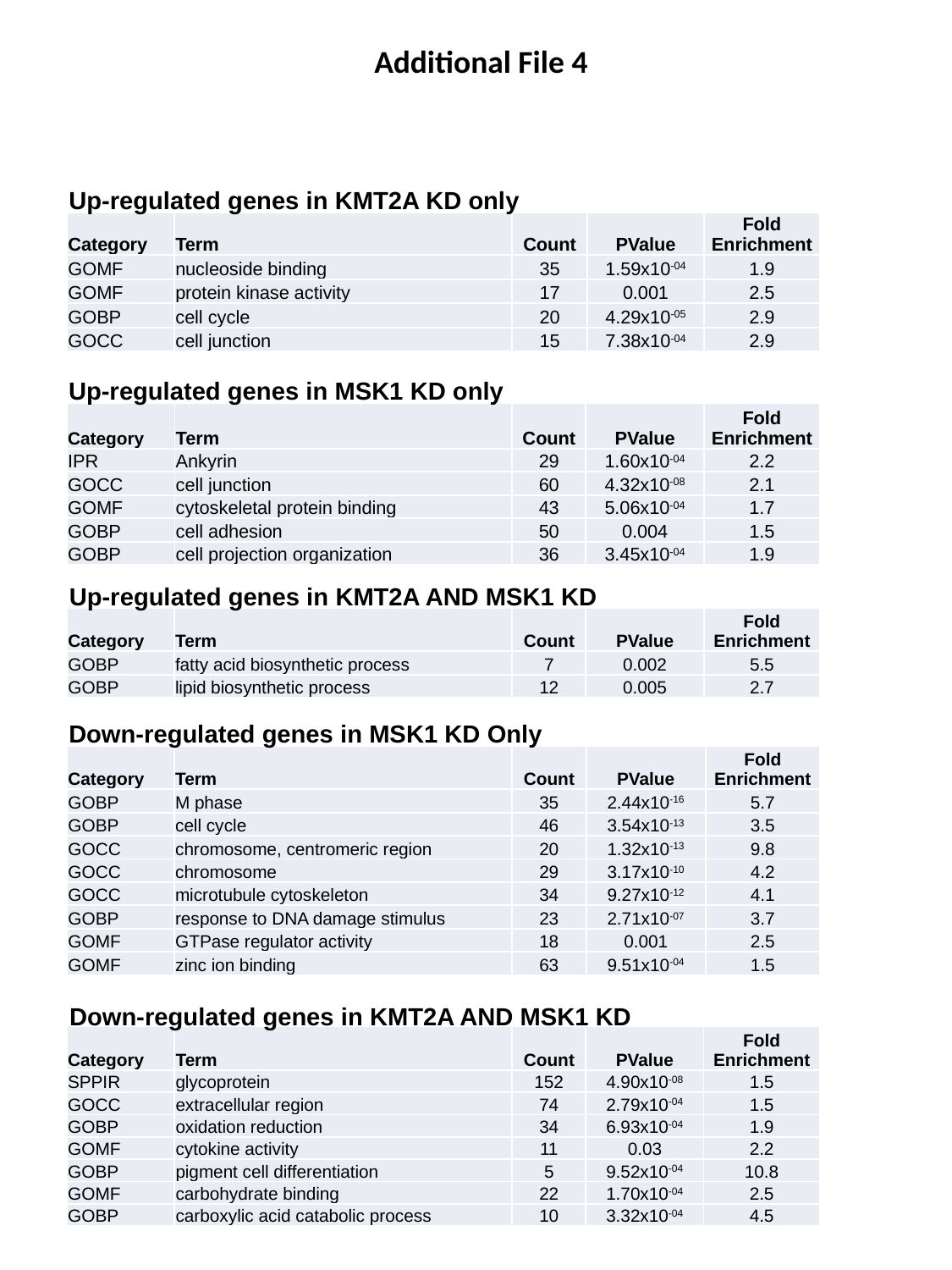

Additional File 4
Up-regulated genes in KMT2A KD only
| Category | Term | Count | PValue | Fold Enrichment |
| --- | --- | --- | --- | --- |
| GOMF | nucleoside binding | 35 | 1.59x10^-04^ | 1.9 |
| GOMF | protein kinase activity | 17 | 0.001 | 2.5 |
| GOBP | cell cycle | 20 | 4.29x10^-05^ | 2.9 |
| GOCC | cell junction | 15 | 7.38x10^-04^ | 2.9 |
Up-regulated genes in MSK1 KD only
| Category | Term | Count | PValue | Fold Enrichment |
| --- | --- | --- | --- | --- |
| IPR | Ankyrin | 29 | 1.60x10^-04^ | 2.2 |
| GOCC | cell junction | 60 | 4.32x10^-08^ | 2.1 |
| GOMF | cytoskeletal protein binding | 43 | 5.06x10^-04^ | 1.7 |
| GOBP | cell adhesion | 50 | 0.004 | 1.5 |
| GOBP | cell projection organization | 36 | 3.45x10^-04^ | 1.9 |
Up-regulated genes in KMT2A AND MSK1 KD
| Category | Term | Count | PValue | Fold Enrichment |
| --- | --- | --- | --- | --- |
| GOBP | fatty acid biosynthetic process | 7 | 0.002 | 5.5 |
| GOBP | lipid biosynthetic process | 12 | 0.005 | 2.7 |
Down-regulated genes in MSK1 KD Only
| Category | Term | Count | PValue | Fold Enrichment |
| --- | --- | --- | --- | --- |
| GOBP | M phase | 35 | 2.44x10^-16^ | 5.7 |
| GOBP | cell cycle | 46 | 3.54x10^-13^ | 3.5 |
| GOCC | chromosome, centromeric region | 20 | 1.32x10^-13^ | 9.8 |
| GOCC | chromosome | 29 | 3.17x10^-10^ | 4.2 |
| GOCC | microtubule cytoskeleton | 34 | 9.27x10^-12^ | 4.1 |
| GOBP | response to DNA damage stimulus | 23 | 2.71x10^-07^ | 3.7 |
| GOMF | GTPase regulator activity | 18 | 0.001 | 2.5 |
| GOMF | zinc ion binding | 63 | 9.51x10^-04^ | 1.5 |
Down-regulated genes in KMT2A AND MSK1 KD
| Category | Term | Count | PValue | Fold Enrichment |
| --- | --- | --- | --- | --- |
| SPPIR | glycoprotein | 152 | 4.90x10^-08^ | 1.5 |
| GOCC | extracellular region | 74 | 2.79x10^-04^ | 1.5 |
| GOBP | oxidation reduction | 34 | 6.93x10^-04^ | 1.9 |
| GOMF | cytokine activity | 11 | 0.03 | 2.2 |
| GOBP | pigment cell differentiation | 5 | 9.52x10^-04^ | 10.8 |
| GOMF | carbohydrate binding | 22 | 1.70x10^-04^ | 2.5 |
| GOBP | carboxylic acid catabolic process | 10 | 3.32x10^-04^ | 4.5 |
