# Supplementary figures and images for: Protein kinase Msk1 physically and functionally interacts with the KMT2A/MLL1 methyltransferase complex and contributes to the regulation of multiple target genes
Source: Epigenetics Chromatin. 2016 Nov 11;9:52. doi: 10.1186/s13072-016-0103-3 (PMC5106815; doi:10.1186/s13072-016-0103-3)

## Slide 1
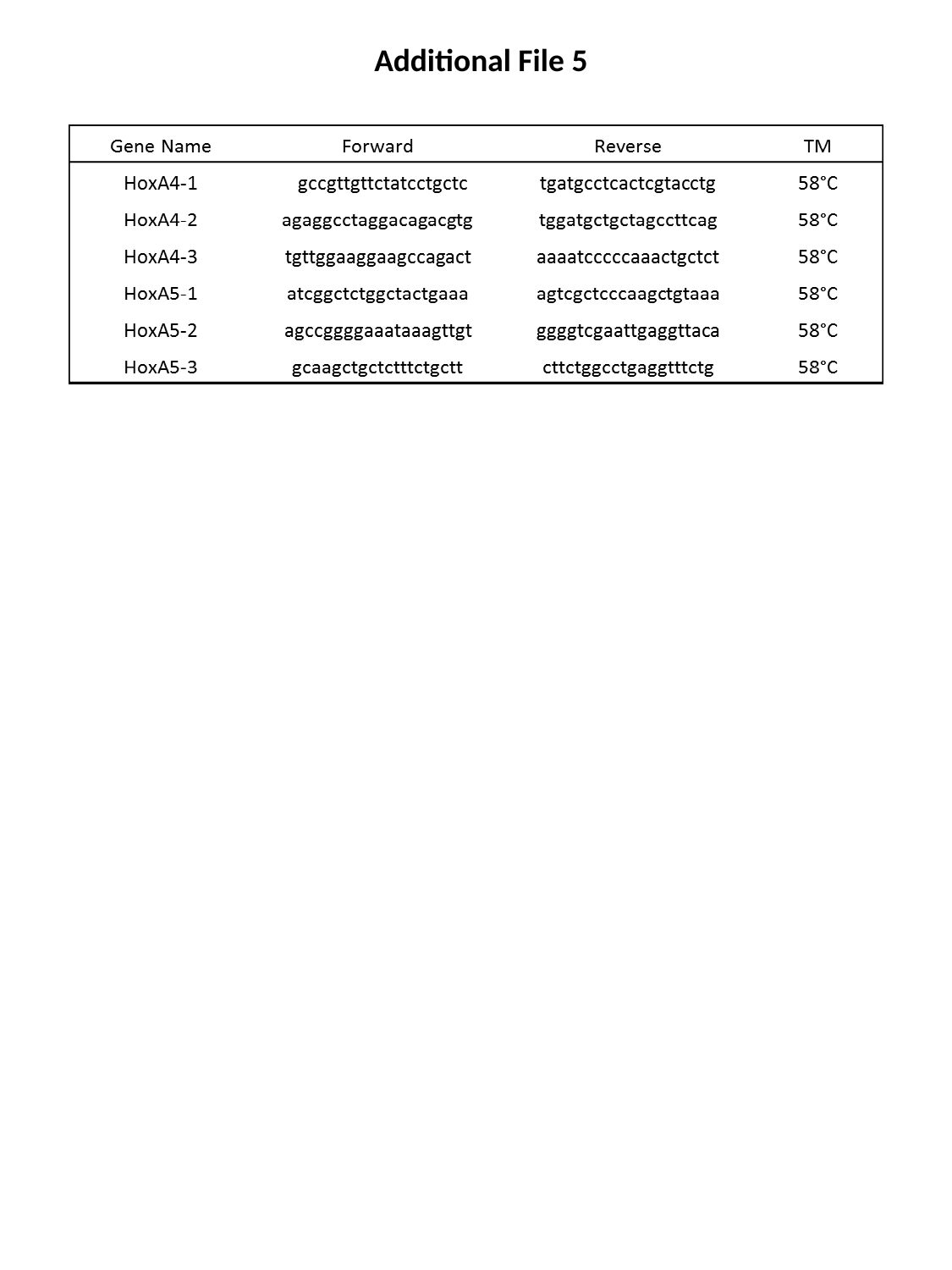

Additional File 5

Supplement: Supplementary file 5 — Additional file 5. qPCR primer sets for C-ChIP and X-ChIP analysis. [file 13072_2016_103_MOESM5_ESM.pptx]
